# A novel ventriculoperitoneal shunt flow sensor based on electrically induced spatial variation in cerebrospinal fluid charge density

**DOI:** 10.3389/fbioe.2023.1339831

**Published:** 2024-01-11

**Authors:** David A. Zarrin, Matiar Jafari, Won Kim, Geoffrey P. Colby

**Affiliations:** ^1^ David Geffen School of Medicine, University of California, Los Angeles, Los Angeles, CA, United States; ^2^ Department of Neurosurgery, University of California, Los Angeles, Los Angeles, CA, United States

**Keywords:** ventriculoperitoneal shunts, cerebrospinal fluid, hydrocephalus, flow sensing, cerebral ventricles

## Abstract

**Introduction:** Ventriculoperitoneal (VP) shunts divert cerebrospinal fluid (CSF) out of cerebral ventricles in patients with hydrocephalus or elevated intracranial pressure (ICP). Despite high failure rates, there exist limited clinically viable solutions for long-term and continuous outpatient monitoring of CSF flow rate through VP shunts. We present a novel, low-power method for sensing analog CSF flow rate through a VP shunt premised on induced spatial electrical charge variation.

**Methods:** Two geometric variants of the proposed sensing mechanism were prototyped: linear wire (P1) and cylindrical (P2) electrodes. Normal saline was gravity-driven through P1 and a commercially available shunt system in series. True flow rates were measured using a high-precision analytical balance. Subsequently, artificial CSF was driven by a programmable syringe pump through P2. Flow rate prediction models were empirically derived and tested. Sensor response was also assessed during simulated obstruction trials. Finally, power consumption per flow measurement was measured.

**Results:** P1 (17 mm long) and P2 (22 mm long) averaged 7.2% and 4.2% error, respectively, in flow rate measurement from 0.01 to 0.90 mL/min. Response curves exhibited an appreciably flattened profile during obstruction trials compared to non-obstructed states. P2 consumed 37.5 µJoules per flow measurement.

**Conclusion:** We propose a novel method for accurately sensing CSF flow rate through a VP shunt and validate this method at the benchtop with normal saline and artificial CSF over a board range of flows (0.01–0.90 mL/min). The sensing element is highly power efficient, compact, insertable into existing shunt and valve assemblies, and does not alter CSF flow mechanics.

## Graphical Abstract

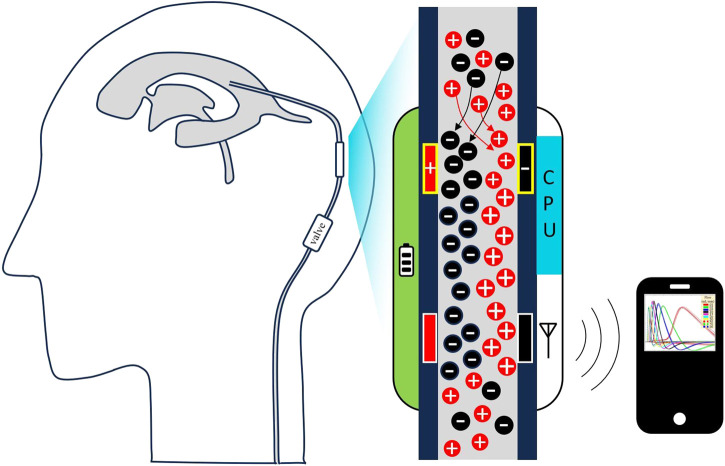

## 1 Introduction

An estimated 30,000 ventriculoperitoneal (VP) shunts are implanted annually in the United States yet as many as 40% fail within 2 years due to flow obstruction, leading to thousands of emergent shunt revisions and a potentially fatal outcome if not promptly addressed (Ahmadvand et al., 2020). The rate of shunt revision is known to be higher than primary implantation, as was recently supported by a nationwide epidemiologic study of VP shunt revision rates between 2008 and 2021 in Norway (Mansoor et al., 2023). Furthermore, there are an even greater, though incompletely documented, number of diagnostic workups for suspected shunt failure in the United States each year. Such workups may consist of an emergency department visit, brain imaging, shunt tapping, and possibly ICP monitoring, altogether easily costing thousands of dollars, and often turning up negative (Sood et al., 2000; March, 2005; Bratton et al., 2007). Numerous mechanisms underlie shunt failure, including shunt obstruction, valve failure, disconnection, catheter fracture, and displacement, among others (Paff et al., 2018). Each of these failure modes ultimately leads to the same endpoint: arrested CSF flow through the shunt and the potential for a subsequent elevation in intracranial pressure (ICP) and irreversible neurologic injury (Desai et al., 2020).

Given flow arrest precedes neurologic decline secondary to shunt malfunction, numerous devices have been developed to track CSF flow rate in VP shunts or predict VP shunt obstruction. The earliest potential solutions came about in the 1980s and relied upon velocity tracking of electrolytically generated bubbles within the CSF, however these solutions were limited by bubble adhesion to catheter walls and their gravity dependent function (Hara et al., 1982; Hara et al., 1983; Numoto et al., 1984). Microelectromechanical systems (MEMS) technology was used by Raj and colleagues to develop a pressure-sensitive flow sensor using a pair of flexible silicone membranes (Raj et al., 2015). This approach offers the advantage of power efficiency and pressure information in addition to flow rates, but it requires the sensing element to extend over 10 cm along the catheter and relies on consistent fluid drag of the inner catheter wall for accurate flow calculation based on membrane deflection. Gamero et al. (2021) developed an intra-abdominal CSF flow sensor based on resistance and pressure with a reported battery life of 7 years, however this method only provides flow measurements during a single 2 min window each day, requires manual use of an external reader each time, and only reported three discrete flow levels rather than a continuous analog scale. Other solutions measure the changing electrical impedance of CSF to assess the degree of patency of the shunt, in particular the number of holes which are patent on the tip of the ventricular catheter (Basati et al., 2015; Kim et al., 2016). Degree of catheter patency is valuable in understanding overall shunt health, but alone is not sufficient due to multiple failure modes being independent of shunt occlusion (disconnection, displacement, fracture, valve failure).

Over the past decade, thermal anemometry has become a popular sensing mechanism utilized in CSF flow monitoring devices due to its reliable and fully analog measurement of flow rate. This method relies on the direct correlation between flow rate and rate of convective heat dissipation from a thermal source. Both transcutaneous and implantable thermal anemometric solutions have achieved accurate measures of CSF flow in benchtop and clinical settings (Bork et al., 2010; Qin et al., 2017; Hudson et al., 2019; Qin et al., 2019; Krishnan et al., 2020), but are dependent on parameters which vary by individual such as skin thickness and site of sensor placement (Gamero et al., 2021). Although accurate, thermal anemometry solutions are limited by their power expensive sensing mechanism, as it requires appreciable energy to repeatedly drive current through a heating element. As a result, any implantable solution based on thermal anemometry requires repeated inductive charging for long-term use, which poses a challenge from a device management standpoint primarily in an outpatient setting.

The above limitations have slowed progress towards the elimination of neurologic morbidity and mortality associated with VP shunt failure. The ideal device would be fully implantable, leverage a low power sensing mechanism to obviate repeated re-charging, and would continuously monitor multiple variables such as analog flow rate, catheter patency, and intraventricular pressure to assess VP shunt health. The convenience of such a solution coupled with the ability to non-invasively detect shunt failure and diagnose the specific cause of shunt malfunction would help to drive clinical adoption.

To move towards the described ideal solution, our group has developed a new method for accurately measuring analog CSF flow rate in a VP shunt (and other CSF diversion systems) which relies on a low power sensing mechanism. The geometry of the sensing element allows for its insertion into existing VP shunt systems during initial shunt placement. The sensor is comprised of conductive electrodes which induce a local change in CSF charge density and subsequently measure a flow rate-dependent response downstream. The study herein describes the sensor’s working principle, design, and benchtop performance for measuring flow rate of both normal saline and artificial CSF (aCSF) through a catheter and commercially available VP shunt and valve assembly.

## 2 Materials and methods

### 2.1 Sensing mechanism

**FIGURE 1:**
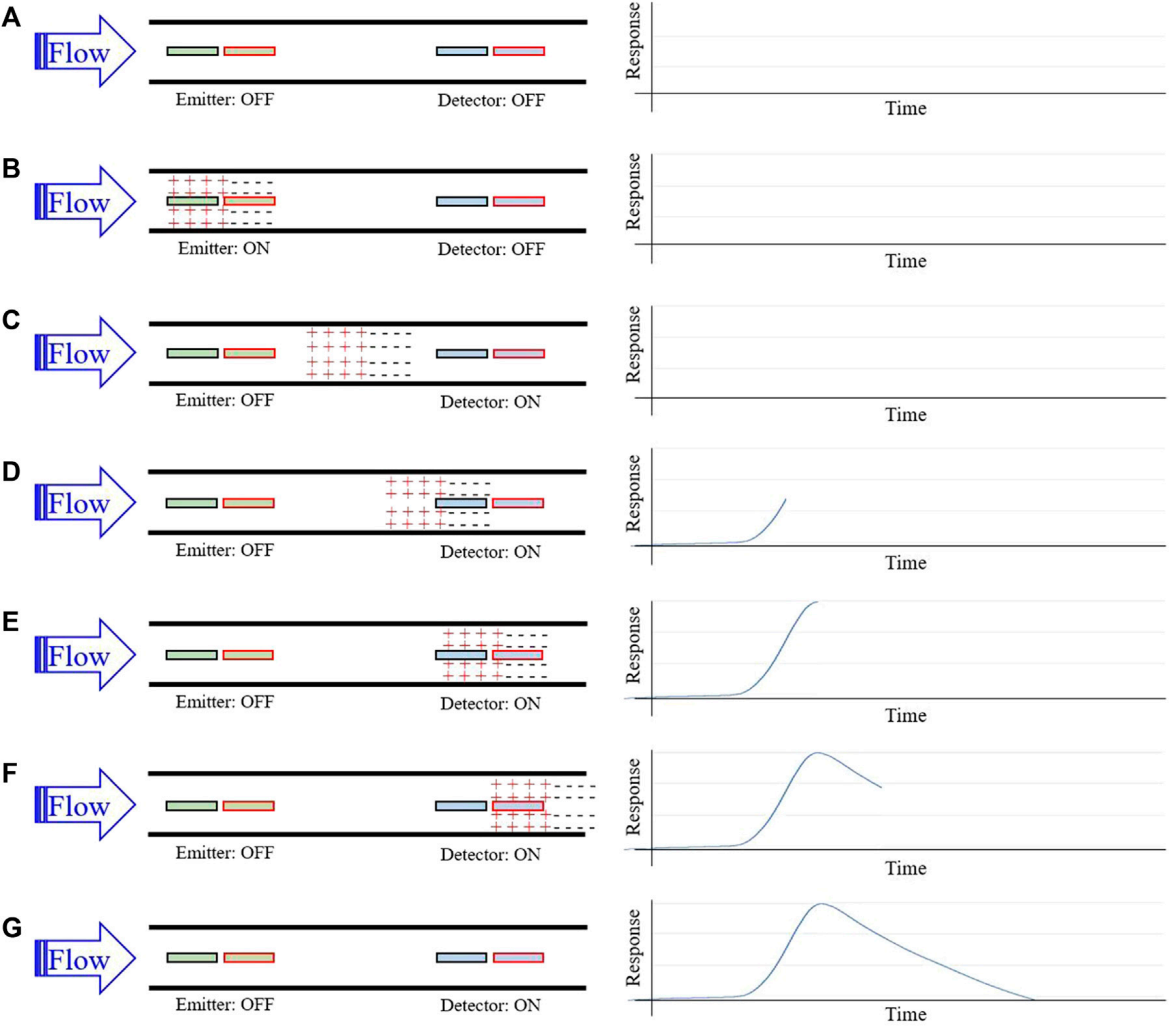
Sensor working principle for flow rate measurement. **(A)** Homogenously charged CSF flows through the catheter and over the emitter and detector; **(B)** A brief voltage pulse applied across the emitter plates induces a positively and negatively charged cloud of ions in local CSF; **(C)** Continued CSF flow carries the charged ion clouds downstream; **(D)** the charged cloud begins to induce a detectable difference in potential across detector plates; **(E)** As the charged clouds pass further over the detector plates, the detected difference in potential maximizes; **(F)** The difference in potential diminishes towards baseline as the charged cloud continues downstream; Flow rate is estimated using time-to-peak of the response signal and the known distance between the emitter and detector plates.

A set of conductive electrodes lines the inner surface of the flow channel within the sensor and electrically interacts with CSF as it flows by. The first two electrodes encountered by CSF comprise the emitter, and the final two electrodes comprise the detector (Figure 1A). When a voltage is applied across the emitter electrodes, a cloud of relative positive charge is formed near the negative electrode, and a cloud of relative negative charge is formed near the positive electrode (Figure 1B). Upon removal of the emitter voltage, ions within each cloud begin to diffuse towards electrical neutrality through Brownian motion. This process takes time and does not complete before the CSF reaches the detector plates when flowing at typical flow rates of 0.01–1 mL/min (Figure 1C). The charged ion clouds then serve as a battery by inducing a measurable voltage across detector electrodes (Figure 1D). This detected voltage first rises, peaks, and then returns to baseline as the charged clouds pass by the detector (Figures 1E, F). Given a known time of emitter stimulation, a known time of arrival at the detectors, and a known distance between the emitter and detector electrodes, a flow rate is determined.

### 2.2 Sensor design

**FIGURE 2:**
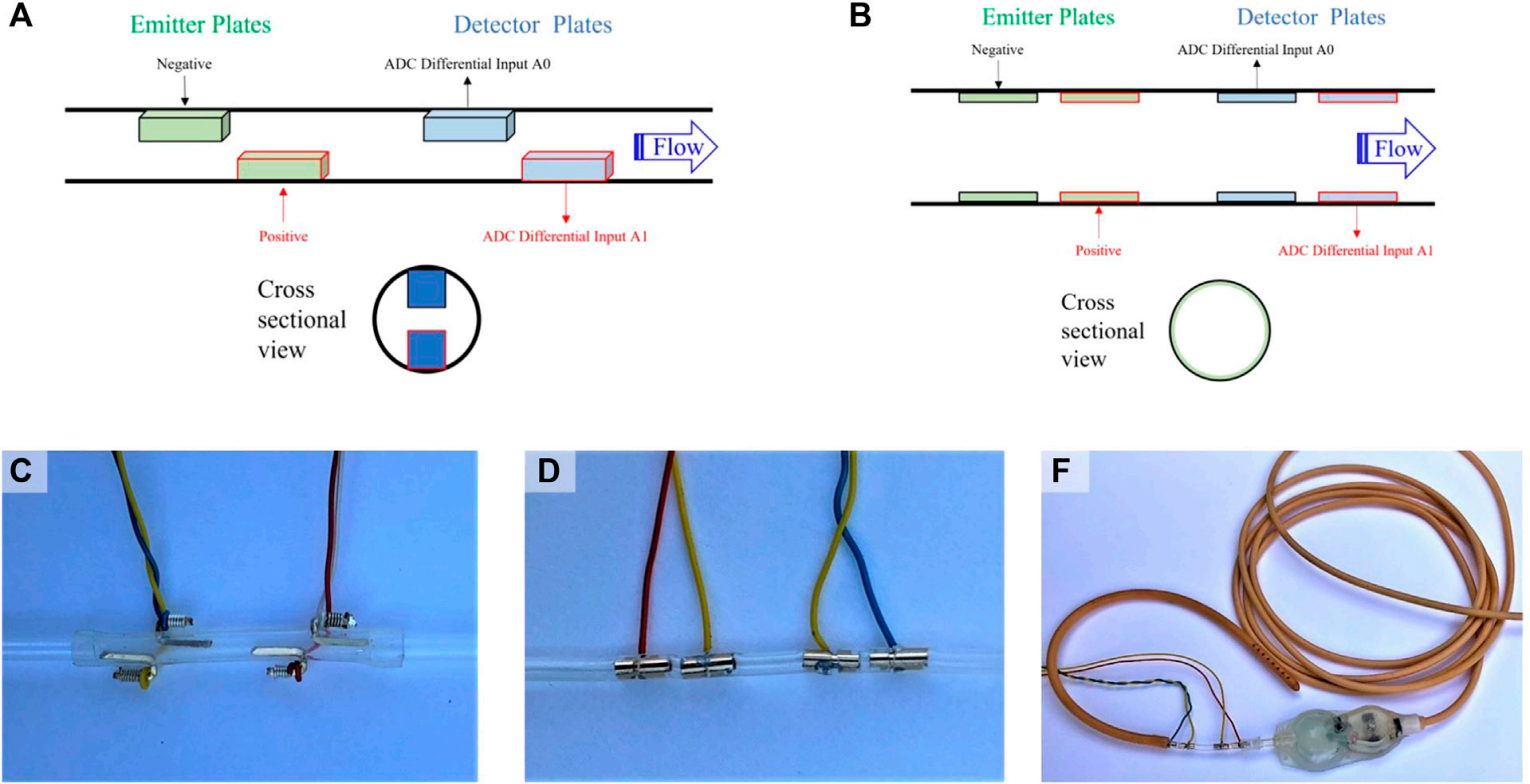
Sensor design and positioning. **(A)** Depiction of the general wire electrode sensor design (P1), which consists of linear conductive wires comprising an emitter and detector. CSF flows from the emitter to the detector; **(B)** Depiction of the general cylindrical electrode sensor design (P2), which consists of conductive cylinders comprising an emitter and detector. CSF flows from the emitter to the detector; **(C)** A photograph of the wire electrode flow sensor; **(D)** A photograph of the cylindrical electrode flow sensor; **(E)** A photograph of the cylindrical electrode flow sensor in its intended mode of operation, namely, inserted between the ventricular catheter and pressure outlet valve of a valve and shunt assembly.

Two electrode geometries were tested in this study, including a wire electrode design (P1, Figure 2A) and a cylindrical electrode design (P2, Figure 2B). The wire electrode design consists of two gold-plated wires oriented parallel to the flow, coincident with the inner catheter wall, axially offset, and diametrically opposed comprising each the emitter and detector (Figure 2C). The cylindrical electrode design consists of two gold-plated cylinders with outer surfaces coincident with the inner catheter wall, comprising each the emitter and detector (Figure 2D). The catheter segment of all sensors was comprised of silicone. The cylindrical electrode flow sensor in its intended mode of operation, inserted between the ventricular catheter and pressure outlet valve of a valve and shunt assembly, is shown in Figure 2E.

### 2.3 Experimental setup

**FIGURE 3:**
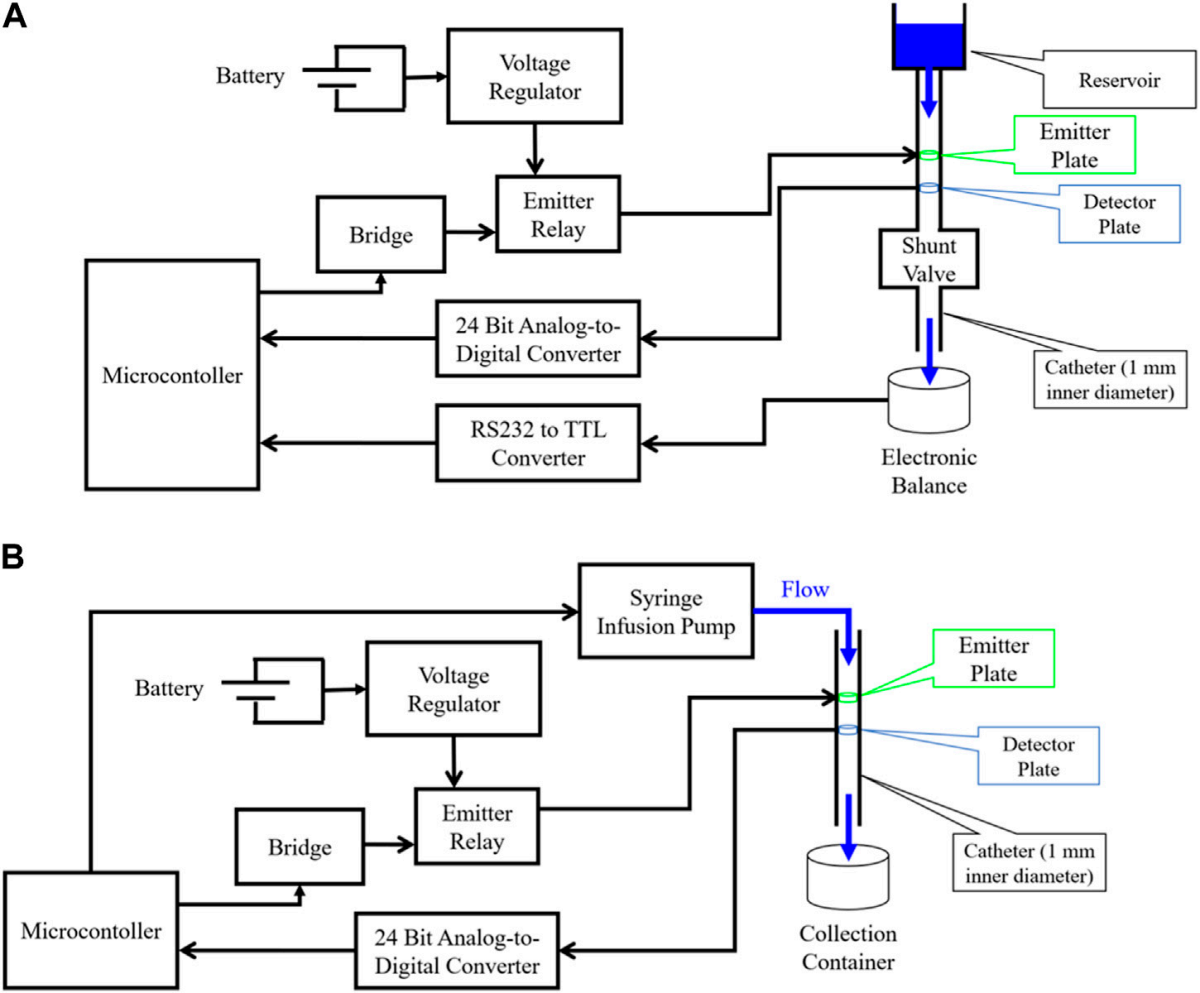
Diagram of experimental setups used for evaluating flow sensor accuracy. **(A)** Gravity driven flow experimental setup. Emitter plates are stimulated by a voltage-regulated power source, and an analog-to-digital converter (ADC) captures analog signals from the detector plates and sends them to digital input/output (I/O) pins of a microcontroller; **(B)** Syringe pump driven flow experimental setup. Emitter plates are stimulated by a voltage-regulated power source, and an ADC captures analog signals from the detector plates and sends them to digital I/O pins of a microcontroller.

For analog flow rate testing, normal saline [0.9% NaCl, *σ* = 1.45 S/m (Sauerheber and Heinz, 2015)] was first driven by gravity drain through a CODMAN**®** CERTAS® Plus Programmable Valve (CODMAN 2023) and Shunt Assembly (Figure 3A) with the P1 placed directly before the valve to evaluate for the presence of the hypothesized flow-dependent voltage response. Once verified, the electrode design was updated to the cylindrical design (P2), which maximizes fluid-to-electrode contact surface area per unit device length. aCSF [Ecocyte Bioscience (EcoCyte Bioscience, 2023), *σ* ∼ 1.4 S/m (Bedard et al., 2022)] was then driven through P2 by a New Era® 1000 One Channel Programmable Syringe Pump (Syringe Pump, 2023) (Figure 3B). In each case, a microcontroller [ATmega328P (ATMEGA328P, 2023)] controlled a bridge which operated the emitter relay, thereby applying a voltage-regulated source to the emitter. In all experiments, analog voltages from the detector were converted to digital signals using a 24-bit analog-to-digital converter (ADC) (ADS1256 data sheet, 2023) and stored in memory by the microcontroller. For gravity-driven flow (Figure 3A), a CGOLDENWALL**®** Precision Digital Analytical Balance (CGOLDENWALL, 2023) measured the mass of drained fluid in real-time and transferred this data to the microcontroller for estimation of true flow rates.

### 2.4 Wire electrode flow sensor testing

The wire electrode flow sensor was tested using normal saline (0.9% NaCl) driven by gravity (Figure 3A). Each gravity drain trial consisted of a several hour-long drain with periodic flow measurements made throughout. Each flow measurement lasted 1 min, beginning with one second of dead time, followed by applying 1.1 V to the emitter for 2 s, followed by 57 s of capturing and storing analog voltages across the detector. The voltage curves captured by the detector in a test set of five gravity drains were then used to develop an algorithm for automatically estimating flow rate based on the detected voltage signal. This algorithm uses peak detection to determine the delay to the maximum (time-to-peak) or minimum (time-to-valley) signal voltage during each minute-long trial. Polynomial fits were established to define a mathematical function relating time-to-peak/valley with true flow rate. The algorithm was then tested on a hold-out set of five separate gravity drain trials, where a prediction of flow rate was made based on each detected voltage curve alone. These predictions were compared against the true flow rates as estimated by data from the analytical balance to evaluate overall sensor performance.

### 2.5 Cylindrical electrode flow sensor testing

A similar process was conducted to test the cylindrical electrode sensor, this time with aCSF driven by the syringe pump (Figure 3B). The aCSF used consisted of physiologic solute concentrations (NaCl = 125 mM, KCl = 3 mM, CaCl2 = 2.5 mM, MgSO4 = 1.3 mM, NaH2PO4 = 1.25 mM, NaHCO3 = 26 mM, Glucose = 13 mM) and was controlled at pH of 7.4 by bubbling the solution with carbogen gas as is standard procedure. The syringe pump was used to drive flow at ten different rates ranging from 0.01 to 0.9 mL/min through the sensor. Ten measurements were made at each flow rate, five for model fitting and five for accuracy evaluation as before. Finally, a manual shunt obstruction during a gravity drain of normal saline through the cylindrical electrode flow sensor was conducted to evaluate the ability of P2 to detect acute shunt obstruction. To achieve manual obstruction, a mechanical vice grip was applied to the external surface of the shunt catheter to fully close the lumen in each manual obstruction trial.

### 2.6 Mathematical modeling

Simple mathematical models were developed to bound the earliest and latest expected voltage change in the detector of P2. All fluids were assumed to be incompressible, and flows were assumed to be steady. Models were derived using the fundamental fluid mechanical continuity equation, which is a statement of conservation of mass (Munson et al., 2012):

$$\rho \left(\frac{\partial }{\partial t}\int_{cv}dV+\int_{cs}V∙\hat{n}\;dA\right)=0$$

where *t* is time, ρ is fluid density, d V is the differential volume, *V* is fluid velocity, *dA* is a differential area, the term $V∙\hat{n}\;dA$ is the volume flow rate through *dA*, and *cv* is the control volume and *cs* is the control surface of a region bounded by the cylindrical space spanning all four electrode contacts in length, concentric with the catheter, and with a radius equal to that of the catheter’s inner diameter. With all fluids assumed to be incompressible and flowing steadily, the first term, (time rate of change of mass within the defined control volume) is zero, and the second term (net rate of mass flow through the control surface) formalizes our assumption of constant flow rate throughout the volume. Time delays to signal detection were then defined as:

$$\left[t_{\min}\left(V_{f}\right),t_{\max}\left(V_{f}\right)\right]=\left[\frac{D_{electrodes,\min\;}}{V_{f}},\frac{D_{electrodes,\max\;}}{V_{f}}\right]$$

where *t*_*min*_ *and t*_*max*_ represent the minimum and maximum delays to signal detection from the time of external voltage application respectively, *V*_*f*_ represents the flow velocity of the fluid, and *D*_*electrodes,min*_ (4 mm) and *D*_*electrodes,max*_ (22 mm) represent the distance between closest and furthest points along the flow path where a fluid molecule may directly contact an electrode, respectively. The first model (*t*_*min*_) effectively assumes that charged fluid at the most downstream point of the emitter electrodes is immediately detected upon arriving at the most upstream point of the detector. Conversely, a second model (*t*_*max*_) assumes that charged fluid at the most upstream point of the emitter is detected only at the most downstream point of the detector. These models therefore bound the expected arrival time of charged fluid at the detector and operate on the assumption that only fluid which directly contacts the emitter during active stimulation can become locally charged.

### 2.7 Secondary sensor characterization

Several additional experiments were conducted using the cylindrical electrode flow sensor to further characterize sensor behavior. The effects of isolated changes to stimulation voltage and duration on the detected signal were studied. Finally, the power and energy requirements of the sensing element were explored by measuring current drawn during the 1.1 V stimulation of the emitter.

### 2.8 Data analysis

All data were analyzed, and graphs were generated using MATLAB (R2022b). Raw voltages were imported into MATLAB and the peakfinder function within the Signal Processing Toolbox was utilized to identify the most prominent peak or valley within each detected voltages signal and the associated time delay. Training set peak and valley time delays were passed into the Polyfit MATLAB function to determine flow rate prediction models. Separate testing set time delays were passed into predictive models and predicted flow rates were compared to true flow rates as determined by preset flow rates in infusion pump experiments, or by linear regression of fluid mass as reported by the analytical balance as a function of time in commercial shunt and valve experiments.

## 3 Results

### 3.1 Wire electrode flow sensor test results

**FIGURE 4:**
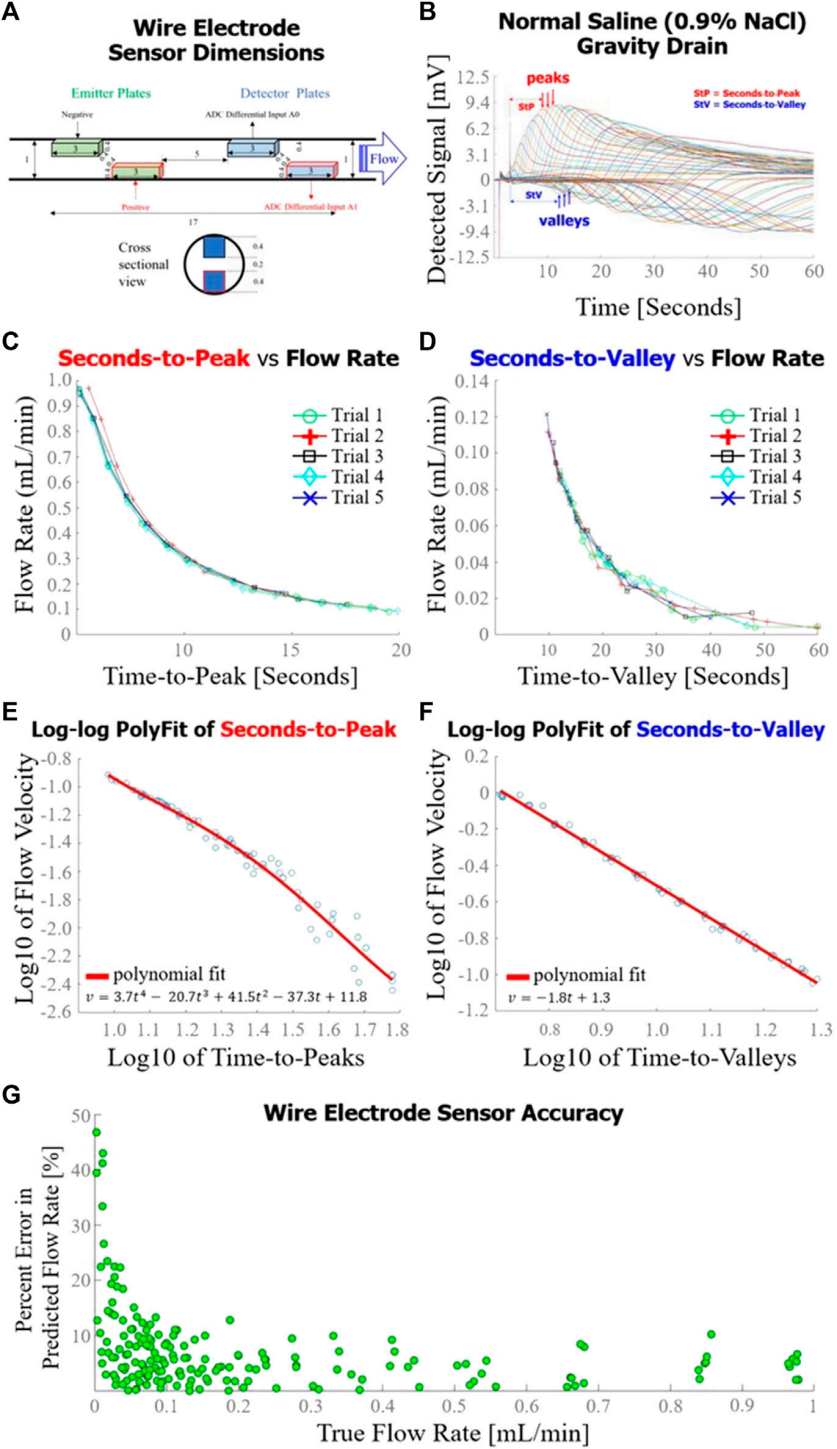
Wire electrode sensor design and performance. **(A)** Dimensions [mm] of the wire electrode flow sensor used in this set of experiments; **(B)** Sensor response curves measured throughout a gravity drain experiment as configured in Figure 2A. Time-to-peak (for high flow rates) or time-to-value (for lower flow rates) were analyzed for each curve to estimate flow rate; **(C)** Flow rate as a function of time-to-peak for five gravity drain trials, each trial consisting of approximately 15 distinct flow rate measurements; **(D)** Seconds-to-valley as a function of flow rate for five gravity drain trials, each trial consisting of approximately 15 flow rate measurements; **(E)** Fourth order polynomial fit of log10 of flow rate as a function of log10 of time-to-peaks; **(F)** First order polynomial fit of log10 of flow rate as a function of log10 of time-to-valleys; **(G)** Overall accuracy of the wire electrode sensor as determined by percent error in predicted flow rate as a function of true flow rate.

The exact dimensions of the wire electrode sensor are detailed in Figure 4A. The voltages detected during a typical gravity drain of normal saline through the wire electrode flow sensor are superimposed in Figure 4B. The hypothesized voltage peaks described in Figure 1 were observed in the detector response, along with the addition of unexpected dips in voltage consistently occurring at lower flow rates of <0.3 mL/min (forming the valleys ultimately used in time-to-valley modeling). Seconds-to-peak (Figure 4C) and seconds-to-valley (Figure 4D) as functions of flow rate were plotted. A fourth order polynomial was fit to the log10 of seconds-to-peak vs. log10 of flow rate (Figure 4E), and a first order polynomial was fit to log10 of seconds-to-valley vs. log10 of flow rate (Figure 4F). The sensor achieved an average percent error of 7.2% in flow rate measurement evaluated against a total of 180 true flow rate measurements over a broad range of flow (0.01–0.9 mL/min) (Figure 4G). For P1, percent error in flow rate measurement was elevated for low flow rates (<0.04 mL/min).

### 3.2 Cylindrical electrode flow sensor test results

**FIGURE 5:**
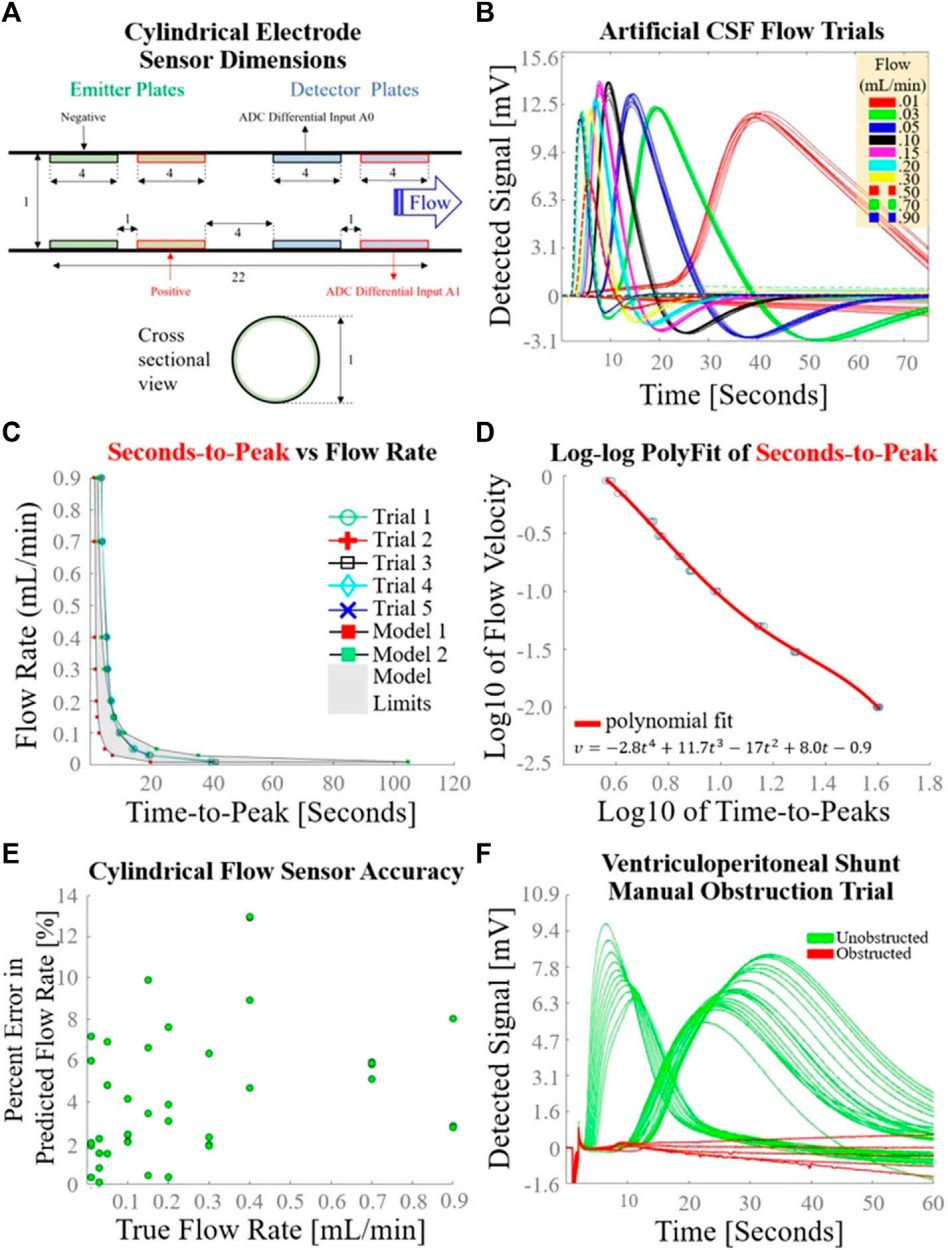
Cylindrical electrode sensor design and performance. **(A)** Dimensions [mm] of the cylindrical electrode flow sensor used in this set of experiments; **(B)** Sensor response curves measured 10 times at each of 10 different syringe-pump driven flow rates, using the experimental setup depicted in Figure 2B. Seconds-to-peak was the feature used to estimate flow rate for each curve; **(C)** Flow rate as a function of time-to-peak for the five trials used to determine a flow estimation model; **(D)** Fourth order polynomial fit of log10 of flow rate as a function of log10 of time-to-peaks; **(E)** Overall accuracy of the cylindrical electrode flow sensor as determined by percent error in predicted flow rate as a function of true flow rate. **(F)** Sensor response during a gravity drain with transient manual catheter obstruction.

The exact dimensions of a longitudinal midline cross-section of the cylindrical electrode flow sensor are detailed in Figure 5A. The voltages detected by the cylindrical electrode flow sensor during the syringe pump-driven aCSF trials are superimposed in Figure 5B. Response curves were in close alignment with the hypothesized voltage peaks described in Figure 1. Seconds-to-peak as a function of flow rate for the five trials used to develop the flow estimation model alongside mathematical models corresponding to earliest theoretical detection time (Model 1) and latest theoretical detection time (Model 2) with intervening area shaded gray are plotted in Figure 5C; the shapes of the empirically and mathematically derived seconds-to-peak vs. flow rate responses mirrored one another as parabolic curves. A fourth order polynomial was fit to log10 of seconds-to-peak vs. log10 of flow rate (Figure 5D). The average percent error of the cylindrical flow sensor using the polynomial fit to generate flow estimations over a flow range of 0.01–0.9 mL/min was 4.2% (Figure 5E). Unlike P1 which had elevated percent error at low flows <0.04 mL/min, P2 maintained relatively constant percent error across all flow rates. Detected voltages for successive measurements during the VP shunt manual obstruction trials are shown in Figure 5F. Unobstructed trials tended to show voltage peaks in the detected signal as observed in other analog flow rate accuracy tests. In contrast, obstructed trials exhibited a flattened profile compared to unobstructed trials, suggesting that the charged ion cloud was not carried to the detector via bulk fluid motion due to the obstructed state of the system.

### 3.3 Secondary sensor characterization results

**FIGURE 6:**
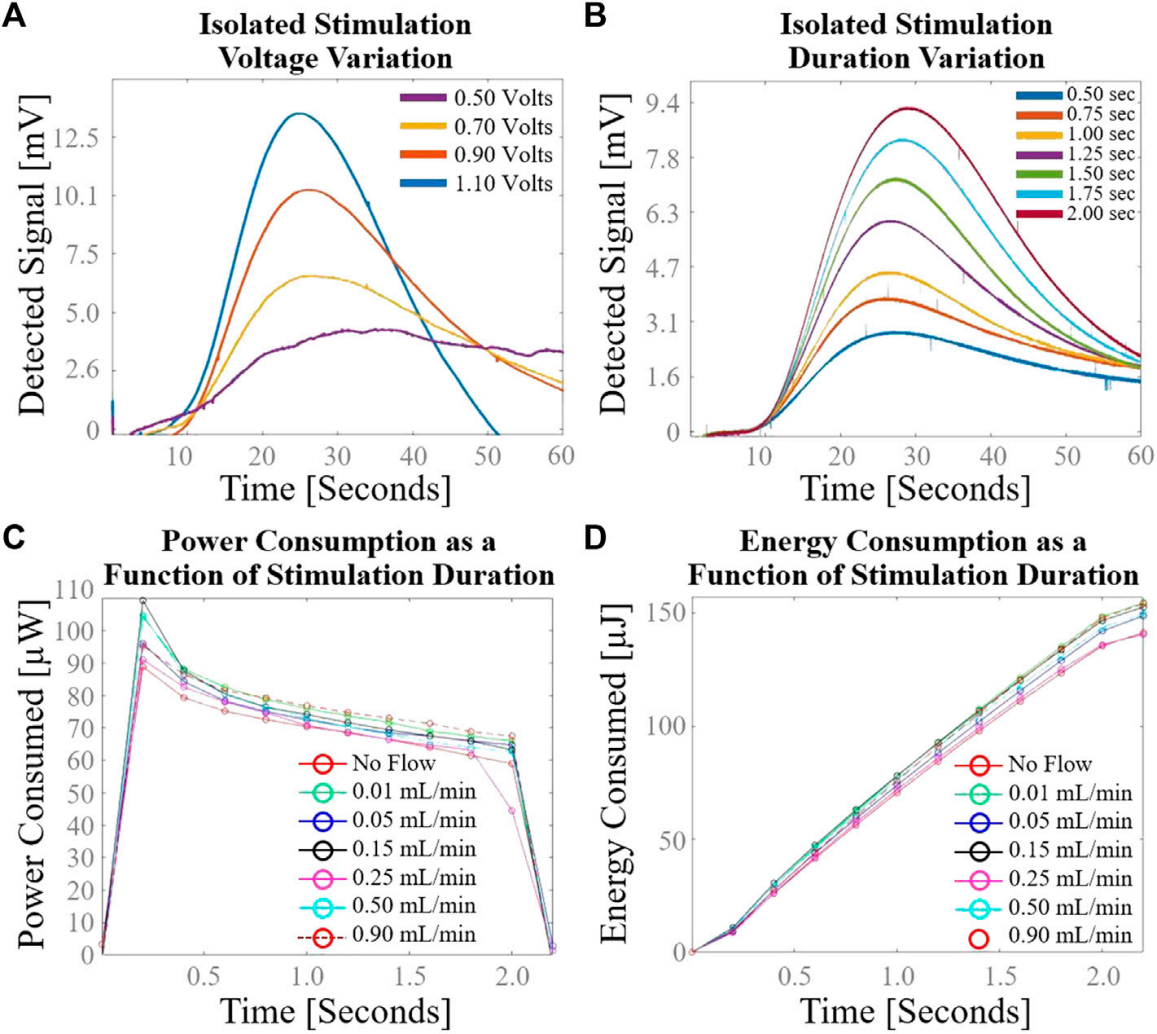
Secondary cylindrical electrode flow sensor characterization using normal saline. **(A)** Variation in sensor response with isolated change to stimulation voltage; **(B)** Variation in sensor response with isolated change to stimulation duration; **(C)** Power consumption as a function of stimulation duration demonstrates capacitance-like behavior with asymptotically decreasing charge rate after initial voltage application; **(D)** Energy consumption as a function of stimulation duration demonstrates the low power requirements of the sensing element.

Detected voltage in response to variation in stimulation voltage at constant flow rate and stimulation duration is shown in Figure 6A. The time-to-peak did not change appreciably with changing stimulation voltage. Signal clarity is compromised at stimulation voltage of 0.50 V with the cylindrical plate design. Detected voltage in response to variation in stimulation duration at constant flow rate and stimulation voltage is shown in Figure 6B. The time-to-peak did not change appreciably with changing stimulation duration. Signal clarity was not comprised as low as 0.50 s stimulation duration, as evidenced by the preservation of a clear signal peak at 0.50 s stimulation duration. Power consumption as a function of stimulation duration at various flow rates of normal saline is shown in Figure 6C. The power curve exhibits capacitance-like behavior with asymptotically decreasing charge rate after initial voltage application. Energy consumption as a function of stimulation duration at various flow rates of normal saline is shown in Figure 6D. Fewer than 40 µJoules are consumed by the sensing element during one flow rate measurement with a pulse duration of 0.5 s.

## 4 Discussion

The proposed sensing element achieved accurate measurements of flow rates ranging from 0.01–0.9 mL/min while consuming less power than direct thermal anemometry solutions by a factor of over 400. The sensing element was also capable of distinguishing obstructed from unobstructed states with a high signal-to-noise ratio. The described flow sensor employs a novel sensing mechanism leveraging the time-dependent dispersion of a charged fluid through Brownian diffusion. The time it takes CSF to flow ∼2 cm through the catheter between the emitter and detector matches the time associated with CSF’s transient return to spatial electrical homogeneity. This congruence in time allows for a viable method to measure flow using our compact sensor design as described above. Due to the combination of CSF’s constant fluid mechanical flow and the partial electrical conductivity of CSF, the fluid between the electrodes effectively functions as a hybrid electrical resistor and capacitor, which allows for some flow of current while also retaining charge after the external voltage is removed. The fluid’s nonzero electrical conductivity allows for sufficient charge separation upon application of the external voltage, allowing for subsequent detection of heterogenous electrical charge at the detector plate. If the fluid were non-conducting, the solution would be inviable because no charge separation would occur. CSF *in vivo* is indeed slightly more conductive (*σ* = 1.8 S/m) (Latikka and Eskola, 2019) than the normal saline (*σ* = 1.45 S/m) and aCSF (*σ* ∼ 1.4 S/m) tested in this study, suggesting sufficient charge separation will be achieved in prospective *in vivo* testing. Because our method involves applying an EM field directly through a segment of CSF, the sensing element saves significantly on power as it only requires 37.5 µJoules per flow measurement. In direct thermal anemometry solutions, the thermistor is placed in direct contact with CSF to minimize transcutaneous heat loss associated with indirect thermal anemometry, making direct solutions more power-efficient per flow measurement than indirect thermal anemometry (Krishnan et al., 2020); yet the direct thermal anemometry solution described in Qin et al. (2017) still required ∼17 millijoules per flow measurement, which is ∼450 times higher than the power required in our present solution.

The proposed flow sensor is capable of reporting accurate measurement in variable conditions in large part due to the implementation of a reference electrode. The emitter effectively serves as a reference for the detector, abstracting away several characteristics of the fluid which are subject to temporal variation. The chemical composition of CSF may change over time as a result of shunt surgery itself (Tullberg et al., 2008) or various pathological conditions, resulting in variable cell counts, the presence or absence of blood, and variable protein, ion, and sugar levels. Despite the significantly differing chemical compositions of normal saline and aCSF, flow rates of both fluids were accurately measured using the described time-to-peak algorithm across typical VP shunt flow rates. Furthermore, no effort was made to control the pre-stimulation state of ionization in the fluid. Given repeatable flow rate sensor accuracy results in this setting, it follows that the pre-existing state of ionization of the fluid does not significantly affect the accuracy of the flow sensor. Unlike existing thermal anemometry solutions, it stands to reason that temperature variation would similarly have negligible effect on the transit time of the ion cloud from emitter to detector. This is unlike thermal anemometry solutions (Bork et al., 2010; Qin et al., 2017; Hudson et al., 2019; Qin et al., 2019; Krishnan et al., 2020), which are inherently temperature dependent. Further experimentation is necessary to confirm the fluid-temperature independent nature of our sensing technology.

We found that electrode geometry influenced the maximum achievable accuracy of flow rate measurement. The averaged percent errors in flow rate measurement for the wire and cylindrical electrode flow sensors were 7.2% and 4.2%, respectively, figures which are comparable to the most accurate current solutions (Qin et al., 2017; Krishnan et al., 2020). These accuracies were tested for flows ranging from 0.01–0.9 mL/min, which covers reported physiologic CSF shunt flow rates (Kadowaki et al., 1995). The physical explanation for the observed difference may relate to the total conducting surface area available for interaction with the fluid. With more conductive surface contacting the fluid in the cylindrical emitter as compared to the wire emitter, the cylindrical emitter likely generated more spatially distinct ion clouds. Similarly, the larger detector surface likely facilitates improved detection of potential differences within the ion cloud, hence improving flow sensor accuracy. Being symmetrical, a cylindrical electrode design flush with the catheter’s inner wall offers the benefit of no alteration in CSF flow mechanics (other than slightly differing drag coefficients of the conducting surface compared to the silicone tubing). Future iterations of this technology may employ a design with one or more emitters and multiple detectors spaced throughout the catheter, therefore allowing for characterization of the full flow profile throughout the catheter.

Several factors influence the ideal stimulus voltage applied by the emitter. Stimulus voltages below 0.7 V resulted in a low detected voltage and a poor signal-to-noise ratio on the detector plate, presumably due to insufficient electrically induced spatial variation in CSF charge density at the emitter. It should be noted that 0.7 V as a lower stimulus voltage threshold is unique to the cylindrical emitter geometry evaluated in this study, because this voltage threshold is associated with a particular geometry-dependent electromagnetic (EM) field formed between the emitter’s positive and negative poles. To avoid unwanted chemical reactivity with the ionic contents of CSF, the upper stimulus voltage threshold lies below the reduction potentials of each ion capable of precipitation upon reduction in CSF (Na+: 2.71 V, K+: 2.93 V, Ca2+: 2.87 V, Mg2+: 2.37 V, Cl−: 1.36 V) and below the standard potential for hydrolysis (1.23 V) (Flowers et al., 2015). While early solutions intentionally created electrolytic bubbles for velocity tracking (Hara et al., 1982; Hara et al., 1983; Numoto et al., 1984), this method for fluid velocity measurement proved challenging with frequent bubble adherence to the catheter wall. A value of 1.1 V was therefore selected for high signal-to-noise ratios without the significant risk of driving ionic reduction and precipitation or hydrolysis.

As a standalone and compact insertable component, the sensing element is compatible with existing commercial shunt systems and can also be used to retrofit previously implanted shunt systems. After implanting the tip of the ventricular catheter into the ventricles, neurosurgeons will connect the sensor such that the emitter is adjacent to the ventricular catheter, and the detector is adjacent to the proximal side of the shunt valve. By orienting the sensor as such, CSF will flow from emitter to detector as intended. Positioning the sensor directly adjacent to the valve lends neurosurgeons ease of access to the sensor insertion site during routine shunt implantation (James et al., 2023). Convenient retrofitting of previously implanted shunt systems will also be possible during revisions with the sensor being directly adjacent to the valve, where the surgeon will have already established surgical access. The novel sensing mechanism allows for a compact 2 cm design which is smaller than pressure sensitive silicone membrane flow sensors which span over 10 cm along the length of the catheter (Raj et al., 2015). Future iterations of this technology may be embedded directly into the wall of the catheter of a smart shunt, which would presumably have other sensory data (patency, pressure) to holistically monitor overall shunt health. Connection of the sensor distal to the valve would also be possible. Because the sensor will be positioned extracranially with all positive and negative electrical poles residing outside of the brain, there will not be any electrical current applied by the device to the brain. We therefore do not anticipate an elevated risk of brain injury due to the extracranially implanted device.

The use of metal electrodes as described in the flow sensing element is associated with several potential limitations. Metals selected for the electrodes must be compatible with radiographic and magnetic resonance imaging to avoid the introduction of significant imaging artifact and device migration or loss of function under the influence of an external EM field. Any of these complications would be an unacceptable clinical outcome, given the potential danger to the patient and compromise of image quality, which provides critical information necessary for optimal medical management of patients with hydrocephalus. Furthermore, any metal immersed in aqueous solution has the potential to corrode. To prevent plate corrosion, all prototyped electrodes were coated in a thin layer of gold (Ellioun et al., 2023). Close inspection of electrode plates of the emitter and detector in normal saline and artificial CSF revealed no corrosion after immersion for over 60 days. Other corrosion resistant metals such as copper (Fateh et al., 2020) would be similarly sensible selections as corrosion-resistant electrode coatings in future iterations of this device.

## 5 Limitations

There are several limitations to this study. First, the flow sensor was validated with normal saline and artificial CSF, but still needs validation with human CSF in both healthy and diseased conditions. Although there is not a significant difference in composition between the laboratory-grade aCSF and human CSF, this is still a necessary next step. Furthermore, all tested flows were laminar, and further testing is necessary to validate sensor function under pulsatile flow conditions. We expect that pulsatile flow will minimally affect performance, as the magnitude of fluid pulsation is small in comparison to the spacing of the emitter and detector in the proposed design. Although we do not expect temperature to affect our sensor’s function based on its sensing mechanism, a formal evaluation of sensor performance at different fluid temperatures is also a helpful next step. Additionally, there are numerous electrode geometries other than cylindrical or linear wire may serve to minimize device size and further increase signal-to-noise ratio, and therefore require further exploration and testing. Finally, testing this solution in long-term implanted conditions (months) to assess for material fatigue or corrosion is necessary to prove the durability of the proposed solution.

## 6 Conclusion

We propose a novel method for sensing the flow rate of CSF in a VP shunt which achieves high accuracy in benchtop testing across a broad flow range of normal saline and artificial CSF (0.01–0.9 mL/min) while using significantly less power than existing solutions. Further, the sensing element is insertable into existing shunt and valve assemblies and does not alter CSF flow mechanics. The described CSF flow rate sensing method offers the opportunity for a power-efficient implantable VP shunt monitor requiring minimal device maintenance. Future research will focus on further validation of this device.

## Data Availability

The raw data supporting the conclusion of this article will be made available by the authors, without undue reservation.
